# Long-Term Outcomes of Transcatheter Versus Surgical Aortic Valve Replacement Among Patients With Prior Mediastinal Radiation

**DOI:** 10.1016/j.jscai.2026.104265

**Published:** 2026-02-20

**Authors:** Hossam Elbenawi, Mahmoud Eisa, Muhammad Eltony, Omar Almaadawy, Ahmed El Shaer, Khaled Elfert, Ramzi Ibrahim, Jeremiah P. Depta, Karim M. Al Azizi, Chien-Jung Lin, Yiannis S. Chatzizisis, Stanislav Henkin, Martha Gulati, Andrew M. Goldsweig, Ayman Elbadawi, Islam Y. Elgendy

**Affiliations:** a Department of Cardiovascular Medicine, Mayo Clinic, Rochester, Minnesota; b Department of Internal Medicine, Rochester General Hospital, Rochester, New York; c Department of Internal Medicine, MedStar Health, Baltimore, Maryland; d Department of Internal Medicine, School of Medicine and Public Health, University of Wisconsin-Madison, Madison, Wisconsin; e Division of Gastroenterology, West Virginia University School of Medicine, Morgantown, West Virginia; f Department of Cardiovascular Medicine, Mayo Clinic, Phoenix, Arizona; g Department of Cardiovascular Medicine, Rochester General Hospital, Rochester, New York; h Department of Cardiology, Baylor Scott & White The Heart Hospital − Plano, Plano, Texas; i Division of Cardiovascular Medicine, St. Louis University, St. Louis, Missouri; j Division of Cardiovascular Medicine, University of Miami Health System, Miami, Florida; k Barbra Streisand Women’s Heart Center, Smidt Heart Institute, Cedars-Sinai Medical Center, Los Angeles, California; l Baim Institute for Clinical Research, Boston, Massachusetts; m Department of Cardiovascular Medicine, Baystate Medical Center, Springfield, Massachusetts; n Division of Cardiology, CHRISTUS Good Shepherd Medical Center - Longview, Texas A&M School of Medicine, Bryan, Texas; o Division of Cardiovascular Medicine, Gill Heart and Vascular Institute, University of Kentucky, Lexington, Kentucky

**Keywords:** aortic stenosis, long-term outcomes, mediastinal radiation, surgical aortic valve replacement, transcatheter aortic valve replacement

## Abstract

**Background:**

Limited data suggest that transcatheter aortic valve replacement (TAVR) is associated with similar short-term outcomes to surgical aortic valve replacement (SAVR) among patients with a history of mediastinal radiation. However, the long-term outcomes of TAVR versus SAVR in this growing patient population remain unknown.

**Methods:**

We identified patients with a history of chest radiation who underwent isolated AVR using the multinational TriNetX database from 2010 through 2025. Propensity score matching was performed to account for differences in baseline characteristics. Outcomes included all-cause mortality, stroke, and all-cause hospitalization at 5 years.

**Results:**

The analysis included 933 patients with prior chest radiation who underwent isolated AVR, of whom 785 (84.13%) underwent TAVR, and 148 (15.86%) underwent SAVR. After propensity score matching, 128 patients were included in each group. In the TAVR group, the mean age was 67.6 ± 10.2 years, and 72.7% were women. In the SAVR group, the mean age was 67.5 ± 8.7 years, and 69.5% were women. There was no statistically significant difference in the rates of all-cause mortality (11.7% vs 12.5%; hazard ratio [HR], 1.044; 95% CI, 0.52-2.12; *P* = .91), stroke (15.6% vs 13.3%; HR, 1.373; 95% CI, 0.72-2.63; *P* = .34), and all-cause hospitalization (56.3% vs 64.1%; HR, 0.935; 95% CI, 0.68-1.29; *P* = .65) between TAVR and SAVR at 5 years.

**Conclusions:**

In this observational analysis from a multinational database of patients with prior mediastinal radiation, TAVR was associated with comparable long-term outcomes compared with SAVR. Further prospective studies are warranted to confirm these findings.

## Introduction

Severe aortic valve stenosis may be a late complication of radiation-induced cardiac fibrosis, affecting approximately 25% of patients who have been exposed to mediastinal radiation.^1^ Patients with severe aortic stenosis and a history of mediastinal radiation exposure may experience significantly higher complications and mortality rates following surgical aortic valve replacement (SAVR) compared with those without prior radiation exposure.^2^ This elevated risk is likely due to radiation-related fibrosis of surrounding tissues (including pulmonary fibrosis) and contributes to the presence of multiple cardiac lesions, all of which increase morbidity and mortality following cardiac surgery.^3^

Irrespective of the surgical risk, transcatheter aortic valve replacement (TAVR) provides an alternative to SAVR for patients with symptomatic severe aortic stenosis^4^, ^5^, ^6^; however, patients with prior mediastinal radiation were largely underrepresented in the pivotal TAVR trials.^7^, ^8^, ^9^ Limited data have suggested that TAVR is associated with favorable short-term mortality and morbidity compared with SAVR among these patients.^10^, ^11^, ^12^ However, the long-term outcomes of TAVR vs SAVR in patients with prior mediastinal radiation remain unknown. To address this knowledge gap, we utilized the TriNetX database to assess long-term outcomes in patients with prior mediastinal radiation who underwent isolated transcatheter or surgical AVR.

## Materials and methods

### Data source and study population

**Figure 1:**
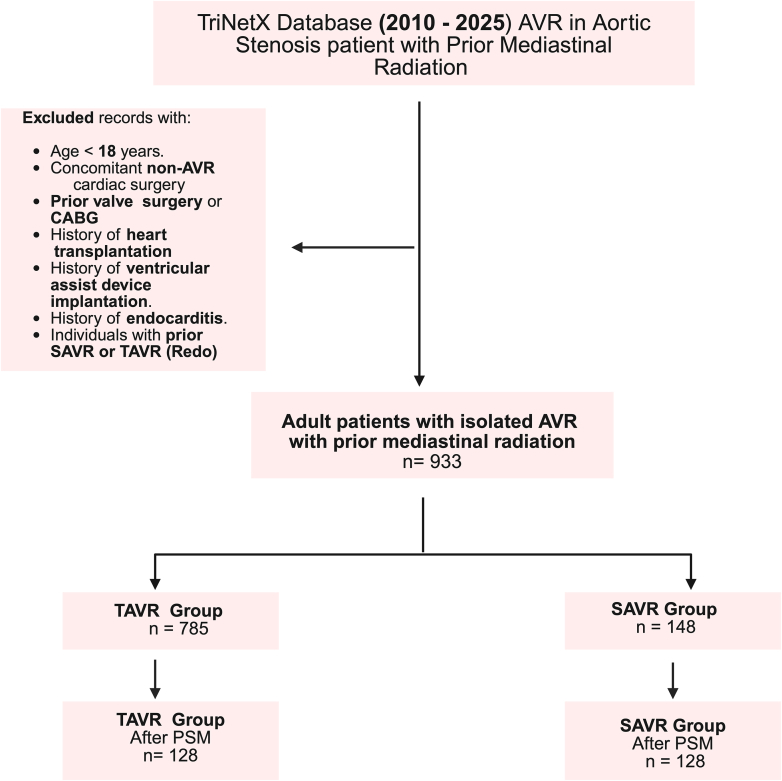
**Study cohort and design reported per the Strengthening of the Reporting of Observational Studies in Epidemiology (STROBE) guidelines.** AVR, aortic valve replacement; CABG, coronary artery bypass grafting; TAVR, transcatheter aortic valve replacement; SAVR, surgical aortic valve replacement; PSM, propensity score matching.

We performed a retrospective, observational analysis using the TriNetX Research Network, which includes electronic health record data from more than 250 million patients across more than 120 healthcare organizations, primarily in the United States.^13^ Within this database, data from 69 US healthcare organizations were queried. Data were deidentified in accordance with the deidentification standard outlined in section x164.514(a) of the Health Insurance Portability and Accountability Act (HIPAA) Privacy Rule. Because this study used deidentified data, the study was exempt from the requirements of the institutional review board. The study was conducted in accordance with the Strengthening the Reporting of Observational Studies in Epidemiology (STROBE) guidelines for observational studies (Figure 1).^14^ The TriNetX database was queried using International Classification of Diseases, Tenth Revision Clinical Modification (ICD-10-CM) Procedure Coding System, Current Procedural Terminology (CPT), and RxNorm codes to identify the study cohort. The inclusion criteria included patients >18 years of age who had nonrheumatic aortic valve stenosis and underwent isolated AVR between January 2010 and January 2025, and had a history of prior mediastinal radiation. Prior mediastinal radiation was defined as a history of irradiation for malignant neoplasms of the breast, esophagus, thymus, heart, pleura, mediastinal lymphoma (including Hodgkin’s, non-Hodgkin’s, follicular, nonfollicular, and other lymphoid types), hematological malignancies, or malignant neoplasms of the bronchus and lung. We excluded patients who underwent concomitant non-AVR cardiac procedures, had a history of prior valve replacement (tricuspid valve, pulmonary valve, or mitral valve) or coronary artery bypass grafting, had a history of heart transplantation or ventricular assist device implantation, had a history of endocarditis, or had previously undergone SAVR or TAVR (redo procedures; Supplemental Appendix S1). We used the CPT code for only transfemoral TAVR. These administrative codes have been previously validated to identify patients with cancer conditions and those with prior radiation history.^15^, ^16^, ^17^ We stratified the cohort into 2 groups based on the modality of AVR:TAVR vs SAVR.

### Study outcomes

The primary outcome was all-cause mortality, whereas the secondary outcomes included all-cause hospitalization and ischemic stroke. Death was handled as a censoring event for nonfatal outcomes. Outcomes were analyzed up to 5 years following the index procedure (TAVR or SAVR).

### Statistical analysis

Continuous variables are presented as mean ± SD and were compared using independent-samples *t* tests. Categorical variables are reported as frequency (percentage) and were compared using the χ^2^ test. To adjust for baseline characteristic differences between the 2 groups, propensity score matching (PSM) was conducted. Covariates were matched 1:1 using the greedy nearest neighbor matching algorithm with a caliper width of 0.1 pooled standardized mean difference (SMD). Any characteristic with an SMD of <0.1 was considered well-matched. The following covariates were included in the PSM: age at index procedure, race, sex, essential hypertension, diabetes mellitus, heart failure, chronic ischemic heart disease, dyslipidemia, history of nicotine dependence, left ventricular ejection fraction, estimated glomerular filtration rate, body mass index, hematocrit, atrial fibrillation and flutter, chronic lower respiratory diseases, carotid artery stenosis or occlusion, peripheral vascular disease, obstructive sleep apnea, use of beta blockers, angiotensin-converting enzyme inhibitors and angiotensin receptor blockers, statins, anticoagulants, and aspirin, and malignant neoplasms of the breast, lung, and lymphoid tissue. Adjusted HRs with 95% CIs were calculated for primary and secondary outcomes.

Cox proportional hazards regression model was conducted to evaluate the association between intervention strategy (TAVR vs SAVR) and long-term all-cause mortality, adjusting for relevant clinical covariates. The model included the following covariates based on clinical relevance and data availability: age at index, sex, race (White), left ventricular ejection fraction (LVEF), estimated glomerular filtration rate, hematocrit, and comorbidities including heart failure, diabetes mellitus, chronic kidney disease, chronic ischemic heart disease, peripheral vascular disease, atrial fibrillation, chronic lower respiratory disease, carotid artery stenosis, sleep apnea, history of smoking, and hematocrit. Cancer history included breast cancer, lung cancer, and lymphoid malignancy. Medication used at baseline, including beta blockers, renin-angiotensin system inhibitors, statins, aspirin, and anticoagulants, was incorporated into the model. Laboratory-based stratifications of LVEF, glomerular filtration rate, and hematocrit were done.

The survival analysis was performed by plotting Kaplan-Meier curves with log-rank tests to compare both cohorts. All tests were 2-tailed; a *P* value of .05 was considered statistically significant without the control of type I error for multiple endpoints. Statistical analyses were performed using the TriNetX online platform with R (Ross Ihaka and Robert Gentleman) for statistical computing. To address the differences in median follow-up time between the groups, time-to-event analyses, including Kaplan-Meier survival curves and Cox proportional hazards models, were employed to account for variable follow-up durations and censoring.

## Results

### Study population and baseline characteristics

A total of 933 patients (63.2% women) underwent isolated AVR across 69 healthcare organizations and were included in the study. Seven hundred eighty-five (84.1%) underwent TAVR, and 148 (15.8%) underwent SAVR. Before PSM, TAVR patients were significantly older than SAVR patients (76.1 ± 9.2 years vs 65.6 ± 9.8 years) and had more comorbidities, including hypertension (85.9% vs 77.0%), diabetes mellitus (41.3% vs 33.1%), chronic ischemic heart disease (82.2% vs 77.7%), heart failure (62.0% vs 42.6%), atrial fibrillation/flutter (35.4% vs 25.7%), smoking history (47.1% vs 31.8%), peripheral vascular disease (16.9% vs 8.8%), and carotid artery stenosis (27.3% vs 25.0%). Additionally, TAVR patients had a lower mean estimated glomerular filtration rate (65.1 ± 22.4 vs 71.2 ± 22.1) and a higher usage of beta blockers (72.5% vs 64.9%), statins (73.9% vs 62.2%), aspirin (78.7% vs 66.2%), and oral anticoagulants (90.1% vs 75%) (Table 1). Malignant neoplasms of the breast did not differ significantly between the 2 groups, although TAVR patients had a higher prevalence of malignant neoplasms of the lung and bronchus (23.8% vs 11.5%, *P* = .001) (Table 1). The propensity score matched 256 patients (128 patients in each group) with well-balanced demographics and baseline characteristics (SMD <0.1 for most covariates). The PSM cohort was comprised of 71.09% women and was predominantly White (89.06%) (Table 1). After propensity score matching, nearly all variables achieved standardized mean differences ≤0.10. LVEF and hematocrit exceeded 0.20, but the absolute differences between groups were small and not clinically meaningful. The median follow-up duration was 775.5 days (IQR, 189-1362) for the TAVR group and 1215 days (IQR, 531-1898) for the SAVR group. The missing data for key variables used in propensity score matching were excluded by the TriNetX platform, ensuring complete case analysis.

**Table 1:** Baseline characteristics before and after propensity score matching.

Variable	Before PSM				After PSM		
	TAVR (n = 785)	SAVR (n = 148)	*P* value	SMD	TAVR (n = 128)	SAVR (n = 128)	SMD
Age, y	76.1 ± 9.2	65.6 ± 9.8	<.001	1.097	67.6 ± 10.2	67.5 ± 8.7	0.013
Female	62.4% (490)	67.6% (100)	.234	0.108	72.7% (93)	69.5% (89)	0.069
White race	84.8% (666)	89.9% (133)	.110	0.152	88.3% (113)	89.8% (115)	0.050
Hypertension	85.9% (674)	77.0% (114)	.007	0.229	79.7% (102)	79.7% (102)	<0.001
Diabetes mellitus	41.3% (324)	33.1% (49)	.063	0.170	37.5% (48)	34.4% (44)	0.065
Heart failure	62.0% (487)	42.6% (63)	<.001	0.397	45.3% (58)	43.8% (56)	0.031
Chronic ischemic heart disease	82.2% (645)	77.7% (115)	.200	0.112	77.3% (99)	79.7% (102)	0.057
Breast cancer	51.3% (403)	54.1% (80)	.544	0.054	56.3% (72)	57.8% (74)	0.032
Lung cancer	23.8% (187)	11.5% (17)	.001	0.328	9.4% (12)	12.5% (16)	0.100
Lymphoid malignancy	29.8% (234)	39.9% (59)	.016	0.212	35.9% (46)	35.9% (46)	<0.001
Peripheral vascular disease	16.9% (133)	8.8% (13)	.012	0.246	8.6% (11)	9.4% (12)	0.027
Atrial fibrillation/flutter	35.4% (278)	25.7% (38)	.022	0.213	31.3% (40)	28.9% (37)	0.051
Chronic lower respiratory disease	45.6% (358)	41.9% (62)	.405	0.075	39.1% (50)	43% (55)	0.079
Smoking history	47.1% (370)	31.8% (47)	.001	0.319	36.7% (47)	36.7% (47)	<0.001
Dyslipidemia	83.9% (659)	77.7% (115)	.064	0.159	80.5% (103)	78.9% (101)	0.039
Carotid stenosis	27.3% (214)	25.0% (37)	.569	0.051	24.2% (31)	24.2% (31)	<0.001
Sleep apnea	21.5% (169)	20.3% (30)	.732	0.031	23.4% (30)	21.9% (28)	0.037
BMI, kg/m^2^	28.9 ± 6.6	31.3 ± 7.3	<.001	0.335	31.1 ± 7.0	30.8 ± 7.3	0.046
Beta-blocking agents	72.5% (569)	64.9% (96)	.06	0.165	66.4% (85)	65.6% (84)	0.016
ACE-I/ARBs	58.7% (461)	53.4% (79)	.227	0.108	55.5% (71)	53.9% (69)	0.031
Statins	73.9% (580)	62.2% (92)	.004	0.253	60.2% (77)	61.7% (79)	0.032
Aspirin	78.7% (618)	66.2% (98)	.001	0.283	71.9% (92)	67.2% (86)	0.102
Anticoagulants	90.1% (707)	75% (111)	<.001	0.405	76.6% (98)	79% (102)	0.076
Left ventricular ejection fraction, %	59.7 ± 12.8	56.9±13.1	.329	0.212	60.3 ± 11.3	57.6 ± 12.9	0.216
Glomerular filtration rate, mL/min/1.73 m^2^	65.1±22.4	71.2 ± 22.1	.003	0.274	68.4 ±23.4	69.7 ±20.2	0.062
Hematocrit	37.5 ± 5.1	38.8 ± 5.3	.006	0.248	37.5 ±5.3	38.8 ±5.2	0.228

Continuous variables are presented as mean ± SD and categorical variables as % (n).

BMI, body mass index; PSM, propensity score matching; SAVR, surgical aortic valve replacement; SMD, standardized mean difference; TAVR, transcatheter aortic valve replacement.

### Outcomes

**Figure 2:**
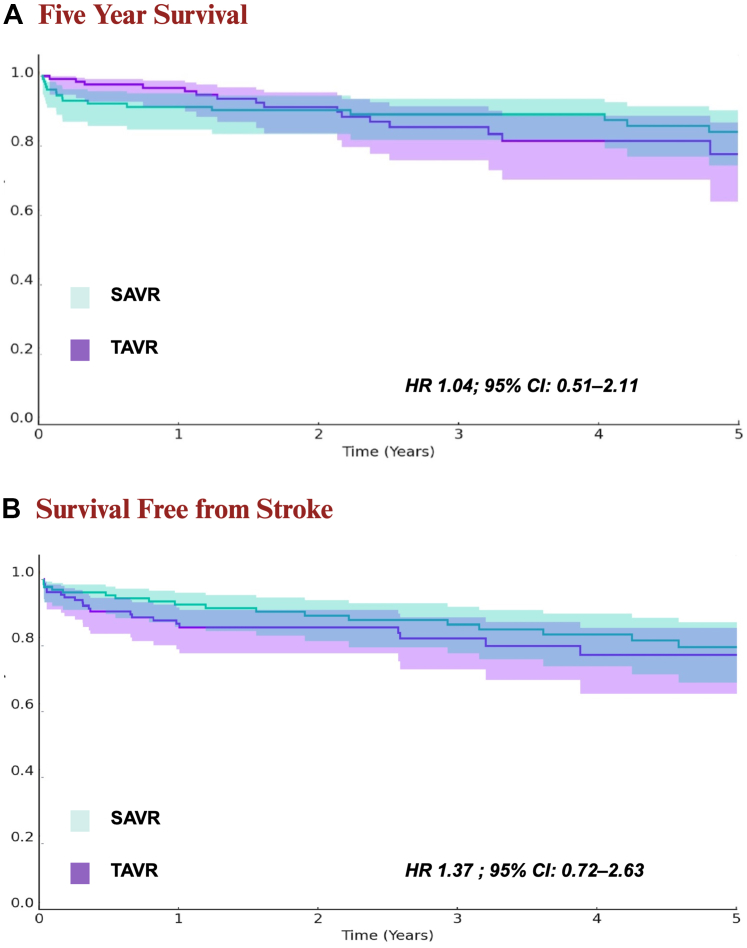
**Five-year outcomes after propensity score matching. (A)** Five-year survival. (**B)** Survival free from stroke. HR, hazard ratio; TAVR, transcatheter aortic valve replacement; SAVR, surgical aortic valve replacement.

For the primary outcome, there was no significant difference in the rate of 5-year mortality between the TAVR and SAVR groups (11.7% vs 12.5%; HR, 1.044; 95% CI, 0.52-2.12; *P* = .91) (Table 2, Figure 2A). For secondary outcomes, there was no significant difference in the rate of all-cause inpatient nonelective hospitalizations between the 2 groups (TAVR vs SAVR: 56.3% vs 64.1%; HR, 0.94; 95% CI, 0.68-1.29; *P* = .65) (Table 2). The rate of ischemic stroke/transient ischemic attack was also similar between the 2 groups (TAVR vs SAVR: 15.6% vs 13.3%; HR, 1.373; 95% CI, 0.72-2.63; *P* = .34) (Table 2, Figure 2B).

**Table 2:** Five-year outcomes before and after propensity score matching.

Outcome	Before PSM					After PSM				
	TAVR (n = 785)	SAVR (n = 148)	HR	95% CI	*P* value	TAVR (n = 128)	SAVR (n = 128)	HR	95% CI	*P* value
Mortality	24.5% (192)	12.2% (18)	2.696	1.65-4.38	.001	11.7% (15)	12.5% (16)	1.044	0.52-2.12	.91
Inpatient nonelective hospitalization	58.5% (459)	62.2% (92)	1.015	0.81-1.27	.889	56.3% (72)	64.1% (82)	0.935	0.68-1.29	.65
Cerebral stroke/TIA	15.5% (122)	14.9% (22)	1.259	0.79-1.98	.320	15.6% (20)	13.3% (17)	1.373	0.72-2.63	.34

HR, hazard ratio; PSM, propensity score matching; SAVR, surgical aortic valve replacement; TAVR, transcatheter aortic valve replacement; TIA, transient ischemic attack.

In the Cox proportional hazard regression model analysis, there was no significant association between treatment cohorts (TAVR vs SAVR) and mortality (HR, 1.517; 95% CI, 0.899-2.561; *P* = .118). Among clinical covariates, history of heart failure was the strongest predictor of mortality with a 2-fold increase in risk (HR, 2.028; 95% CI, 1.435-2.867; *P* < .001). Similarly, a history of lung cancer caused a roughly 2-fold increase in mortality risk (HR, 1.959; 95% CI, 1.277-3.008; *P* = .002). Advancing age was also independently associated with higher mortality, as with every additional year of age, risk increases by 2.3% (HR, 1.023 per year; 95% CI, 1.006-1.041; *P* = .010). Overall, after adjustment for different demographic, clinical, and treatment covariates, heart failure, lung and bronchus cancer, and age were the only independent covariates associated with a change in the risk of mortality in patients undergoing AVR (Table 3).

**Table 3:** Cox proportional hazards model for 5-year mortality.

Covariate	Hazard ratio	95% CI	*P* value
Cohort (TAVR vs SAVR)	1.517	0.899-2.561	.118
Heart failure	2.028	1.435-2.867	<.001
Diabetes mellitus	0.896	0.660-1.216	.482
Age	1.023	1.006-1.041	.010
Peripheral vascular disease	0.868	0.591-1.275	.471
Breast cancer	0.869	0.549-1.375	.549
Lymphoid malignancy	1.160	0.777-1.731	.469
Lung cancer	1.959	1.277-3.008	.002
LVEF 0%-30%	2.073	0.717-5.988	.178
LVEF 30%-50%	1.456	0.854-2.484	.168
LVEF >50%	1.329	0.966-1.829	.080
CKD	1.053	0.726-1.525	.786
Male sex	1.431	0.970-2.112	.071
COPD	1.165	0.849-1.598	.345
White race	0.798	0.522-1.220	.298
Hypertension	1.631	0.973-2.732	.063
Atrial fibrillation	1.138	0.844-1.536	.397
Smoking history	0.831	0.597-1.158	.274
Dyslipidemia	0.932	0.595-1.459	.758
Carotid stenosis	0.816	0.588-1.134	.226
Sleep apnea	0.952	0.672-1.349	.782
Beta blockers	1.176	0.802-1.725	.406
RAAS inhibitor	0.886	0.650-1.206	.441
Statin	0.910	0.620-1.333	.627
Aspirin	1.239	0.809-1.896	.324
Anticoagulant	1.803	0.918-3.542	.087
GFR 0-30, mL/min/1.73 m^2^	1.405	0.948-2.083	.090
GFR 30-45, mL/min/1.73 m^2^	1.103	0.728-1.671	.645
GFR 45-60, mL/min/1.73 m^2^	0.864	0.594-1.255	.442
GFR >60, mL/min/1.73 m^2^	0.980	0.626-1.535	.930
Hematocrit 0%-25%	1.236	0.874-1.749	.230
Hematocrit 25%-35%	1.035	0.711-1.505	.859
Hematocrit >35%	0.645	0.387-1.075	.093
Chronic ischemic heart disease	0.866	0.546-1.373	.540

CKD, chronic kidney disease; COPD, chronic obstructive pulmonary disease; GFR, glomerular filtration rate; LVEF, left ventricular ejection fraction; RAAS, renin-angiotensin-aldosterone system; SAVR, surgical aortic valve replacement; TAVR, transcatheter aortic valve replacement.

## Discussion

To the best of our knowledge, this study is the largest study comparing long-term outcomes of isolated TAVR and SAVR among patients with prior mediastinal radiation. In this observational analysis, we demonstrated that there was no significant difference in 5-year mortality between propensity score-matched patients treated with TAVR or SAVR. Similarly, there were no differences in all-cause inpatient nonelective hospitalizations and ischemic stroke/transient ischemic attack (Central Illustration).

**Central Illustration:**
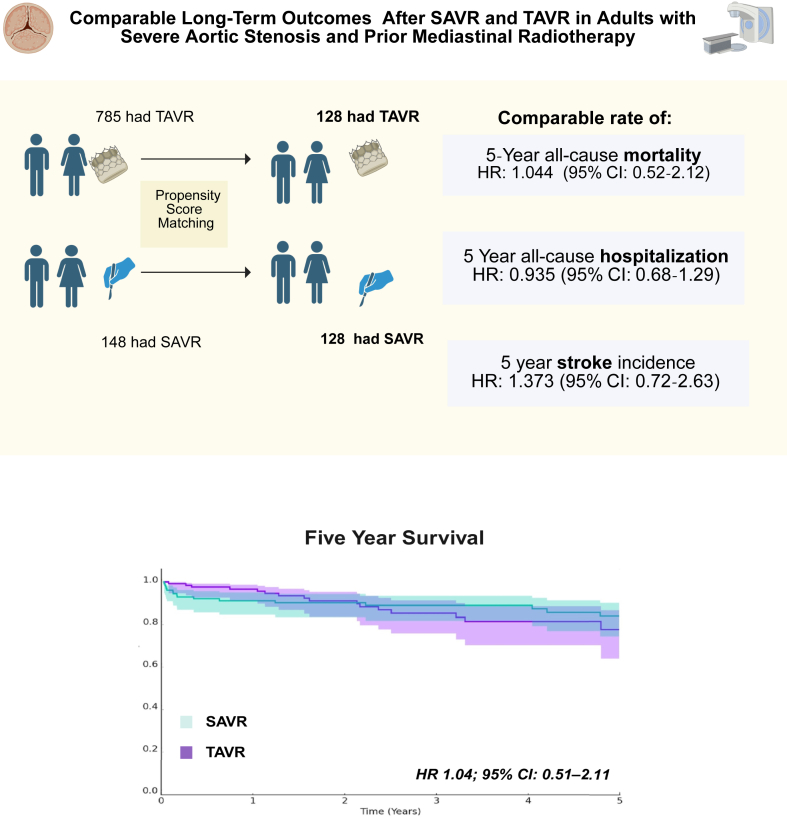
**Long-term outcomes after transcatheter aortic valve replacement (TAVR) versus surgical aortic valve replacement (SAVR) in adults with severe aortic stenosis and prior mediastinal radiotherapy.** HR, hazard ratio.

Previous studies suggested that patients with a history of mediastinal radiation therapy are more likely to have severe coronary artery disease, conduction abnormalities, valvular heart disease, pulmonary disease, and peripheral arterial disease.^2^^,^^18^ In our study, TAVR patients had a higher prevalence of comorbidities compared with SAVR patients. They were older and had a higher prevalence of hypertension, diabetes, atrial fibrillation, peripheral vascular disease, chronic kidney disease, chronic ischemic heart disease, heart failure, and lung cancer. Together, these findings suggest that patients with prior mediastinal radiation present with greater complexity, and those with the highest burden of comorbidity were more likely to be referred for TAVR.

Long-term occurrence of cardiovascular adverse effects associated with cancer treatments, including radiation-related therapies, has garnered significant interest as cancer survival outcomes have improved in recent years.^19^ Despite the high prevalence of aortic stenosis in patients with mediastinal radiotherapy,^1^ there are only a limited number of studies comparing TAVR and SAVR in that patient population, particularly with a focus on long-term outcomes. Our study revealed that the mortality rates for TAVR and SAVR were comparable at 5 years. Similarly, Yazdchi et al^20^ conducted a nonmatched study on a smaller cohort of 69 TAVR and 117 SAVR patients with aortic stenosis associated with prior mediastinal radiotherapy. Their findings demonstrated that TAVR exhibited a similar survival to SAVR at 48 months postoperatively. Additionally, Donnellan et al^21^ reported in-hospital, 1-year, and 2-year survival rates of 96%, 91%, and 86% for TAVR, compared with 96%, 86%, and 80% for SAVR. These findings collectively suggest that TAVR and SAVR offer comparable long-term survival outcomes in these patient populations.

Significant valve fibrosis, calcification, and vascular calcification, commonly seen in radiation-associated aortic stenosis, may elevate the risk of embolization.^22^ This risk could be further amplified with TAVR due to catheter manipulation and transcatheter valve deployment, which can disrupt native valve structures. Despite these concerns, our analysis demonstrated that TAVR was not associated with a higher incidence of stroke compared with SAVR. Although previous studies have established similar rates of short-term perioperative stroke,^10^, ^11^, ^12^ the present study extended our knowledge by showing comparable stroke rates between TAVR and SAVR over a 5-year follow-up period in patients with prior mediastinal radiotherapy. Although the rates of stroke during follow-up in this analysis were higher than prior RCTs of TAVR vs SAVR, our cohort exclusively included patients with prior mediastinal radiation who are at higher risk of stroke compared with the general population.^23^^,^^24^

Controversy regarding readmission rates after TAVR in patients with prior mediastinal radiotherapy had been established in the previous studies; Nauffal et al^12^ showed that there was a comparable rate of 30-day readmission after TAVR or SAVR, and readmission rates of the TAVR group were 10.9% compared with 11.2% for the SAVR group. On the other hand, Zhang et al^11^ revealed that there was a higher readmission rate in the TAVR group compared with the SAVR group on 30 and 90 days (30 days: TAVR 10.9% vs SAVR 5.5%; 90 days: TAVR 30.9% vs 5.5% SAVR). That may be because the TAVR group had more elderly patients, had higher rates of atrial fibrillation, peripheral arterial disease, congestive heart failure, and chronic lung disease. In addition, our matched analysis confirmed that there was no significant discrepancy in the 5-year rate of readmission.

Due to the lack of a randomized controlled trial directly comparing TAVR and SAVR in patients with mediastinal radiation and symptomatic severe aortic stenosis, this observational comparative study, along with others,^10^, ^11^, ^12^^,^^20^ offers some insights to address this knowledge gap.

### Limitations

This is a nonrandomized, observational study. Although a propensity score–based analysis was used to balance patient characteristics between the 2 groups, selection bias related to treatment assignment and residual confounding cannot be ruled out. The significant imbalance in group sizes (785 patients in the TAVR group vs only 148 in the SAVR group) raises concerns about the representativeness and the robustness of findings related to the SAVR cohort. Although matching led to equal sizes for comparison, the smaller SAVR sample may lack statistical power. Additionally, after matching, the TAVR group was younger and had fewer cases of heart failure, peripheral vascular disease, and lung cancer than it did before matching. This indicates that matching selected a lower-risk subpopulation of the TAVR population, limiting the study’s representativeness and generalizability. Data on complex anatomical features—such as bicuspid aortic valve, calcium scores, or hostile or porcelain aorta, which could complicate procedural management—are also not available in the data set. The study does not provide other relevant clinical data, such as the severity of aortic stenosis (including peak and mean gradients and aortic valve area), presence of other valvular diseases, or coexisting pulmonary hypertension. Unfortunately, given the study design, we could not comment on the outcomes of untreated patients with either TAVR or SAVR. Furthermore, patients with higher comorbidity burdens were more likely to undergo TAVR, and the use of older-generation TAVR devices during the study period may have influenced outcomes. Although the study evaluates 5-year outcomes, it lacks detailed information on the surveillance methods used and does not assess long-term complications or postprocedural quality of life. The use of TriNetX data limits adjustment for clustering, as the platform does not support multilevel modeling or generalized estimating equations, leaving potential residual confounding from site-level variation. The nature of the TriNetX database limited our ability to classify patients based on surgical risk. Nevertheless, matching was performed using variables included in the Society of Thoracic Surgeons risk score. Due to platform limitations, deaths were treated as censoring events rather than competing risks, which may underestimate the true incidence of nonfatal outcomes. Additionally, in our analysis, permanent pacemaker implantation occurred in very few patients below the minimum reportable threshold required by TriNetX. Therefore, we were unable to present these results.

## Conclusion

In this multicentral observational study, we found no statistically significant differences in long-term outcomes between TAVR and SAVR in patients with prior mediastinal radiation. However, the study was likely underpowered to detect modest but clinically meaningful differences. Larger prospective studies are needed to better inform clinical decision-making in this high-risk population.

## Footnotes

To access the supplementary material accompanying this article, visit the online version of the *Journal of the Society for Cardiovascular Angiography & Interventions* at 10.1016/j.jscai.2026.104265.
