# Supplementary material for: Long-Term Outcomes of Transcatheter Versus Surgical Aortic Valve Replacement Among Patients With Prior Mediastinal Radiation
Source: J Soc Cardiovasc Angiogr Interv. 2026 Feb 20;5(3):104265. doi: 10.1016/j.jscai.2026.104265 (PMC13005395; doi:10.1016/j.jscai.2026.104265)
Supplement: Supplementary Material [file mmc1.docx]

| **Criteria** | **Category** | **Description** | **Code** |
| --- | --- | --- | --- |
| **Inclusion** | **Diagnosis (Radiation History and Associated Conditions)** | Personal history of irradiation | ICD-10-CM Z92.3 |
|  |  | Malignant neoplasm of breast | ICD-10-CM C50 |
|  |  | Malignant neoplasm of esophagus | ICD-10-CM C15 |
|  |  | Malignant neoplasm of thymus | ICD-10-CM C37 |
|  |  | Malignant neoplasm of heart, mediastinum, and pleura | ICD-10-CM C38 |
|  |  | Hodgkin lymphoma | ICD-10-CM C81 |
|  |  | Follicular lymphoma | ICD-10-CM C82 |
|  |  | Non-follicular lymphoma | ICD-10-CM C83 |
|  |  | Other specified and unspecified types of non-Hodgkin lymphoma | ICD-10-CM C85 |
|  |  | Other and unspecified malignant neoplasms of lymphoid, hematopoietic, and related tissue | ICD-10-CM C96 |
|  |  | Malignant neoplasm of bronchus and lung | ICD-10-CM C34 |
|  | **Procedure (Required for Inclusion)** | Transcatheter aortic valve replacement (TAVR/TAVI) with prosthetic valve; percutaneous femoral artery approach | CPT 33361 |
|  | **Surgical Aortic Valve Interventions** | Replacement, aortic valve, open, with cardiopulmonary bypass; with allograft valve (freehand) | CPT 33406 |
|  |  | Replacement, aortic valve, open, with cardiopulmonary bypass; with stentless tissue valve | CPT 33410 |
|  |  | Replacement, aortic valve; with aortic annulus enlargement, noncoronary sinus | CPT 33411 |
|  |  | Replacement, aortic valve; with transventricular aortic annulus enlargement (Konno procedure) | CPT 33412 |
|  |  | Valvuloplasty, aortic valve, open, with cardiopulmonary bypass | CPT 1029692 |
|  |  | Replacement, aortic valve, open, with cardiopulmonary bypass | CPT 1029693 |
|  |  | Replacement, aortic valve, with cardiopulmonary bypass (deprecated 2018) | CPT 1006147 |
|  |  |  |  |
| **Exclusion** | **Diagnosis (Conditions Leading to Exclusion)** | Endocarditis, valve unspecified | ICD-10-CM I38 |
|  |  | Heart transplant status | ICD-10-CM Z94.1 |
|  |  | Endocarditis and heart valve disorders in diseases classified elsewhere | ICD-10-CM I39 |
|  | **Procedures (Leading to Exclusion)** | Open aortic valve replacement with cardiopulmonary bypass using prosthetic valve | CPT 33405 |
|  |  | Insertion of ventricular assist device | CPT 1006349 |
|  |  | Coronary artery bypass, vein only; single coronary venous graft | CPT 33510 |
|  |  | Coronary artery bypass, vein only; 2 coronary venous grafts | CPT 33511 |
|  |  | Coronary artery bypass, vein only; 3 coronary venous grafts | CPT 33512 |
|  |  | Coronary artery bypass, vein only; 4 coronary venous grafts | CPT 33513 |
|  |  | Coronary artery bypass, vein only; 5 coronary venous grafts | CPT 33514 |
|  |  | Coronary artery bypass, vein only; 6 or more coronary venous grafts | CPT 33516 |
|  |  | Coronary artery bypass, using arterial graft(s); single arterial graft | CPT 33533 |
|  |  | Coronary artery bypass, using arterial graft(s); 2 coronary arterial grafts | CPT 33534 |
|  |  | Coronary artery bypass, using arterial graft(s); 3 coronary arterial grafts | CPT 33535 |
|  |  | Coronary artery bypass, using arterial graft(s); 4 or more coronary arterial grafts | CPT 33536 |
|  |  | Coronary artery bypass, using venous graft(s) and arterial graft(s); single vein graft | CPT 33517 |
|  |  | Coronary artery bypass, using venous graft(s) and arterial graft(s); 2 venous grafts | CPT 33518 |
|  |  | Coronary artery bypass, using venous graft(s) and arterial graft(s); 3 venous grafts | CPT 33519 |
|  |  | Coronary artery bypass, using venous graft(s) and arterial graft(s); 4 venous grafts | CPT 33521 |
|  |  | Coronary artery bypass, using venous graft(s) and arterial graft(s); 5 venous grafts | CPT 33522 |
|  |  | Coronary artery bypass, using venous graft(s) and arterial graft(s); 6 or more venous grafts | CPT 33523 |
|  |  |  |  |
|  | **Other Exclusionary Procedures** | Replacement of Mitral Valve with Nonautologous Tissue Substitute, Percutaneous Approach | ICD-10-PCS 02RG3KZ |
|  |  | Replacement of Mitral Valve | ICD-10-PCS 02RG |
|  |  | Replacement of Pulmonary Valve | ICD-10-PCS 02RH |
|  |  | Replacement of Tricuspid Valve | ICD-10-PCS 02RJ |
|  |  |  |  |
|  |  |  |  |

**Baseline characteristics**

| **Category** | **Description** | **Code** |
| --- | --- | --- |
|  | Essential (primary) hypertension | ICD-10-CM I10 |
|  | Diabetes mellitus | ICD-10-CM E08-E13 |
|  | Heart failure | ICD-10-CM I50 |
|  | Chronic ischemic heart disease | ICD-10-CM I25 |
|  | Malignant neoplasm of breast | ICD-10-CM C50 |
|  | Malignant neoplasm of bronchus and lung | ICD-10-CM C34 |
|  | Malignant neoplasms of lymphoid, hematopoietic, and related tissue | ICD-10-CM C81-C96 |
|  | Peripheral vascular diseases | ICD-10-CM I73 |
|  | Atrial fibrillation and flutter | ICD-10-CM I48 |
|  | Chronic lower respiratory diseases | ICD-10-CM J40-J4A |
|  | Personal history of nicotine dependence | ICD-10-CM Z87.891 |
|  | Disorders of lipoprotein metabolism and other lipidemias | ICD-10-CM E78 |
|  | Occlusion and stenosis of carotid artery | ICD-10-CM I65.2 |
|  | Sleep apnea | ICD-10-CM G47.3 |
|  | Beta-blocking agents | ATC C07 |
|  | Agents acting on the renin-angiotensin system | ATC C09 |
|  | HMG CoA reductase inhibitors (Statins) | ATC C10AA |
|  | Aspirin | NDC 1191 |
|  | Anticoagulants | ATC BL110 |
| **Laboratory Values** | Left Ventricular Ejection Fraction (LVEF) (%) | LOINC 2003 |
|  | Glomerular filtration rate (GFR) | LOINC 8001 |
|  | Hematocrit [Volume Fraction] of Blood | LOINC 9013 |
|  | Body Mass Index (BMI) | LOINC 9083 |
|  |  |  |
| **Outcomes** | Postprocedural cerebrovascular infarction following other surgery | ICD-10-CM I97.821 |
|  | Transient cerebral ischemic attack, unspecified | ICD-10-CM G45.9 |
|  | Transient cerebral ischemia | ICD-9-CM 435 |
|  | Cerebral infarction | ICD-10-CM I63 |
|  | Acute myocardial infarction (AMI) | ICD-10-CM I21 |
